# Dietary patterns derived using principal component analysis and associations with sociodemographic characteristics and overweight and obesity: A cross-sectional analysis of Iranian adults

**DOI:** 10.3389/fnut.2023.1091555

**Published:** 2023-04-17

**Authors:** Sara Ebrahimi, Rebecca M. Leech, Sarah A. McNaughton, Morteza Abdollahi, Anahita Houshiarrad, Katherine M. Livingstone

**Affiliations:** ^1^Institute for Physical Activity and Nutrition, School of Exercise and Nutrition Sciences, Deakin University, Geelong, VIC, Australia; ^2^Social Determinants of Health Research Center, Shahid Beheshti University of Medical Sciences, Tehran, Iran; ^3^Department of Nutrition Research, School of Nutrition Sciences and Food Technology, National Nutrition and Food Technology Research Institute, Shahid Beheshti University of Medical Sciences, Tehran, Iran

**Keywords:** dietary patterns, principal component analysis, obesity, Iranians, adults

## Abstract

**Introduction:**

This study examined the cross-sectional association between household dietary patterns and sociodemographic characteristics and BMI in a nationally representative sample of Iranian adults.

**Methods:**

Data on 6,833 households (*n* = 17,824 adults) from the National Comprehensive Study on Household Food Consumption Pattern and Nutritional Status 2001–2003 were used. Principal component analysis (PCA) was used to extract dietary patterns from three household 24-h dietary recalls. Linear regression analyses were used to examine associations between dietary patterns and sociodemographic characteristics and BMI.

**Results:**

Three dietary patterns were identified: the first was characterized by high citrus fruit intake, the second by high hydrogenated fats intake and the third by high non-leafy vegetables intake. The first and third patterns were associated with household heads with higher education and living in urban areas, while the second was associated with household heads with lower education and living in rural areas. All dietary patterns were positively associated with BMI. The strongest association was found with the first dietary pattern (β: 0.49, 95% CI: 0.43, 0.55).

**Discussion:**

While all three dietary patterns were positively associated with BMI, the sociodemographic characteristics of Iranian adults who consumed them differed. These findings inform the design of population-level dietary interventions to address rising obesity rates in Iran.

## Introduction

Middle-Eastern countries, such as Iran, have faced an accelerated nutrition transition over recent decades (1). Consequently, shifts in dietary patterns from traditional Iranian diets towards Western diets high in added sugars and fat have resulted in higher rates of diet-related chronic diseases and obesity (1, 2). According to the latest available national estimates in 2016, the prevalence of obesity and overweight/obesity among Iranian adults was 23 and 59%, respectively (3). As a poor diet is a significant risk factor for obesity (4) and diet consists of a combination of foods and nutrients, (5) it is crucial to understand the factors that influence overall dietary patterns, such as sociodemographic characteristics, and their associations with overweight and obesity. This will inform the design of strategies to address the health impact of the Iranian nutrition transition.

In our previous study of a nationally representative sample of Iranian adults, the use of overall diet quality indices was shown to be appropriate for use in the population (6–8). However, as Iran is a Middle-Eastern county with a culturally-specific diet, (1) data driven approaches, such as principal component analysis (PCA), present an opportunity to explore the patterns of dietary intake that exist in the population, which diet quality indices may not adequately capture (9–11). PCA has previously been used to assess the dietary patterns of Iranian adults, (12–14) with most identifying an increasingly westernized dietary pattern. For example, in a sample of 10,693 Iranian adults from Yazd, a large urban city, a dietary pattern was identified that was characterized by high intakes of confectionary, sugars and snacks, (12) while findings from a cross-sectional analysis of 4,834 Iranian adults identified a Western dietary pattern, a fast-food dietary pattern and an animal fat dietary pattern (15).

Little is known about the data-driven dietary patterns of Iranians and their associations with sociodemographic characteristics and overweight and obesity (16–19). Research to date has shown mixed associations with BMI, (12, 15) and evidence is inconsistent for the relationship between empirical dietary patterns and sociodemographic characteristics, including age (12, 17, 20, 21) and education (12, 15, 17, 20, 21). Further, no studies have investigated associations with area of residence. Previous research has been conducted in populations from large Iranian cities, such as Shiraz, (22) Isfahan (15, 21) and Tehran, (17, 23) thus limiting the ability to identify culturally specific dietary patterns at a national level and by key sociodemographic characteristics, such as sex and area of residence. Since Iran has experienced rising rates of obesity and urbanization due to the nutrition transition, (2) and obesity is more prevalent in Iranian females than males, (24, 25) understanding the effects of sex and area of residence on the association between dietary patterns and overweight and obesity is of significance. Thus, this study aimed to examine the associations between empirical dietary patterns, sociodemographic characteristics, and overweight and obesity in the National Comprehensive Study on Household Food Consumption Pattern and Nutritional Status 2001–2003. As a secondary aim, this study investigated interactions by sex and area of residence on associations between dietary patterns and overweight and obesity.

## Methods

### Subjects and study design

Dietary, demographic, and anthropometric data were used from 7,248 households (36,014 individuals) from the National Comprehensive Study on Household Food Consumption Pattern and Nutritional Status 2001–2003 (26). Participants from all provinces of Iran (28 at that time) were recruited using a cluster sampling method between March 2001 and November 2003. Trained nutritionists working in the health sector collected information on dietary intake, sociodemographic characteristics, and anthropometry (27). Participants were excluded from the present analysis if (i) they were aged <18 years and (ii) any member of the household reported implausible body weight or height (as detailed later), (iii) households with 0 or >1 custodian to maximize comparability between household dietary recalls. Ethics for the study was approved by the Ethics Committee of Shahid Beheshti University of Medical Sciences and was exempted by the Deakin University Human Research Ethics Committee (reference number 2019–288). This manuscript is reported according to the Strengthening the Reporting of Observational Studies in Epidemiology—Nutritional Epidemiology (STROBE-Nut) reporting checklist (Supplementary Table 1) (28).

### Study measures

#### Dietary intake

Three 24-h dietary recalls were used to collect data on household food and beverage intake over consecutive days (27). The household member who was responsible for cooking in the household provided these data. Households with complete data for 2 days (129 households) or 3 days (7,119 households) of these 24-h dietary recalls were included in the survey. The dietary data were collected in every province throughout the year (except for Ramadan and New Year public holiday period) to capture seasonal variation in dietary intake. Variation was also captured throughout the week by including week days and weekends (27). Dietary data were collected in the household using the *per capita* approach. This dietary assessment methodology considers the equal distribution of intake of food between all members in the household (29). Dietary surveys in low-middle-income countries often collect data at the household-level due to the high cost and complexity of dietary surveys at the individual-level (30–32). To calculate the total food intake per day *per capita*, the total grams of the food consumed in each meal was divided by the number of persons present (including visitors). The intake for each food in each meal was then summed up to calculate the total food intake (g/day/capita) (27). The interviews to collect dietary intake were conducted at the house of the participants. The interviewers used the conversion rate shown on the labels of food products (created by Food and Drug Administration of Iran). For example, for liquid vegetables oils, 1.1 ml was used to convert from volume (cc) to weight (grams). For milk, 1 ml was considered as the conversion rate. Average food and nutrient intake of the 2 or 3 days of 24-h dietary recalls was calculated. An estimate of the ratio of household energy intake to household energy requirement was calculated to adjust for energy intake misreporting. The Iranian Food Composition Dataset was applied to evaluate food items and their energy (kcal/day) and nutrient content. Nutrients examined included sodium (mg/day), fiber (g/day), iron (mg/day), calcium (mg/day), vitamin C (mg/day), and total fat (% of energy), protein (% of energy), and carbohydrate (% of energy) (27). The Iranian Food Composition Dataset was used to evaluate the average content of energy of food groups contributed in PCA to evaluate the percentage of energy contribution from each food groups.

#### Principal component analysis

Dietary data were used to derive dietary patterns using PCA, using varimax rotation. Consistent with the PCA methodology, dietary patterns were constructed based on linear combinations of the food groups, reflecting combinations of foods frequently consumed together. The dietary pattern scores thus indicate the degree to which the participant’s diet conforms to the pattern (33). A total of 43 food groups were created for use in PCA analyses (34). These groupings and the number of food groups were based on previous dietary pattern studies in Iranian adults and the similarity of the nutrient composition of foods, and were pilot tested to avoid low proportions of consumers in each food group (16–19). Input variables for the food groups were grams *per capita* per day. A list of the food groups used is provided in Supplementary Table 2.

The number of dietary patterns was identified based on factors with eigenvalues >1.5, breaks in the scree plots and the interpretability and meaningfulness of the patterns using previous Iranian surveys (16–19). Patterns were described in relation to the food groups that loaded most positively or negatively (factor loading >0.20) for the respective patterns. A factor loading of >0.20 was selected based on previous dietary pattern research in Iranian populations (17–19). The Stata postestimation predict command was used to generate standardized dietary pattern scores.

#### Sociodemographic characteristics

Information on age, sex, education and area of residence were collected during in person interviews with each individual in the household. Area of residence was assessed based on location data provided by the National Statistics Centre, which was used to classify households as living in a rural or urban area (binary variable). For the purpose of regression analyses with household dietary patterns, age and education at the household level were created for the household head (household custodian). Age of adults, and the household heads, was categorized into 18 to ≤40 years (young adults), >40–≤60 years (mid-aged adults), >60 years (older adults) based on previous Iranian research on obesity (35, 36). Education level of adults, and household heads, was categorized into three levels based on the total number of the years of education: low (0–5 years; equivalent to completed primary school), moderate (6–12 years; equivalent to completed secondary school), and high (more than 12 years; equivalent to university education).

#### Anthropometric characteristics

Body weight and height were measured for individuals in households. Weight was measured without shoes and twice to the nearest 100 g using a Seca scale and the mean of these values was used. The accuracy of the scale was adjusted with a control scale (5 kg). Height was measured without shoes and to the nearest 0.1 centimeter with tape measures. Adults with biologically implausible weight (<24.9 or >453.6 kg) and biologically implausible height (<111.8 or >228.6 cm) were excluded from the analysis based on established cut points (37). Body Mass Index (BMI; weight kg/height m^2^) was calculated for every adult in the household. Using the World Health Organization (WHO) cut points, adults were grouped into non-overweight/obese (<25 kg/m^2^) or overweight/obese (≥25 kg/m^2^) (38).

### Data analysis

Data analysis was performed using Stata (version 16.0; StataCorp). The number and percentage of participants were reported for categorical variables and means and standard deviations were reported for continuous variables. The Xtile command was used to create tertiles of dietary patterns. To be able to easily quantify and understand the underlying composition of the dietary patterns, unadjusted linear regression analysis was used to examine nutrient intake across tertiles of each of the three dietary patterns. Furthermore, to evaluate the healthfulness of the identified dietary patterns, the Healthy Eating Index (HEI) and Diet Quality Index International (DQI-I) diet quality indices were examined across tertiles of the dietary patterns. The calculation of these indices has been described in full elsewhere (6, 7). The margins command was used to report the adjusted mean (SD) of food group intakes used in the PCA analysis. Linear regression analysis was used to assess how household dietary patterns (continuous dependent variables) varied across household sociodemographic characteristics (independent variables): the age of household heads (categorical), sex of household heads (binary), education level of household heads (categorical), and area of residence (binary). Multi-level linear regression analysis was used to assess how individual-level BMI (continuous dependent variable) varied by dietary patterns of households (continuous independent variable). Multi-level logistic regression analysis was used to assess the association between household dietary patterns (continuous independent variable) and individual-level obesity (binary and dependent variable). Confounders were identified using a Directed Acyclic Graph (DAG) and previous research (Supplementary Figure 1). DAGs are visual tools used to better understand the relationship between exposures, outcomes and potential confounders (39). A DAG was used to identify the confounders for the association between dietary patterns and overweight/obesity, where age, sex, education, area of residence and energy intake were identified as confounders available for use in this study. Analyses for the associations with BMI and overweight/obesity were adjusted for age (continuous), sex (binary), education (categorical), area of residence (binary) and energy intakes in adults. To examine interactions by sex and area of residence, interaction terms were added to the regression models. Considering the lack of available data on physical activity, and that 50% of the Iranian adult population has insufficient physical activity (40) a sensitivity analysis was conducted to examine whether adjusting analyses for the ratio of energy intake: energy requirement (instead of adjusting for energy intake) changed the direction and strength of associations. As this study used dietary data collected at the household level, a further sensitivity analysis was conducted to examine if adjusting for household size changed the direction or strength of the association between household dietary patterns and adults’ individual-level BMI. Household size was operationalized as no children (<18 years), less than three children and more than three children. The number of children was based on the 1996 Iranian census - the latest census in Iran at the time of the survey (41). A complete case analysis approach was used to manage any missing data. *p* value < 0.05 was considered to determine statistical significance.

## Results

**Figure 1:**
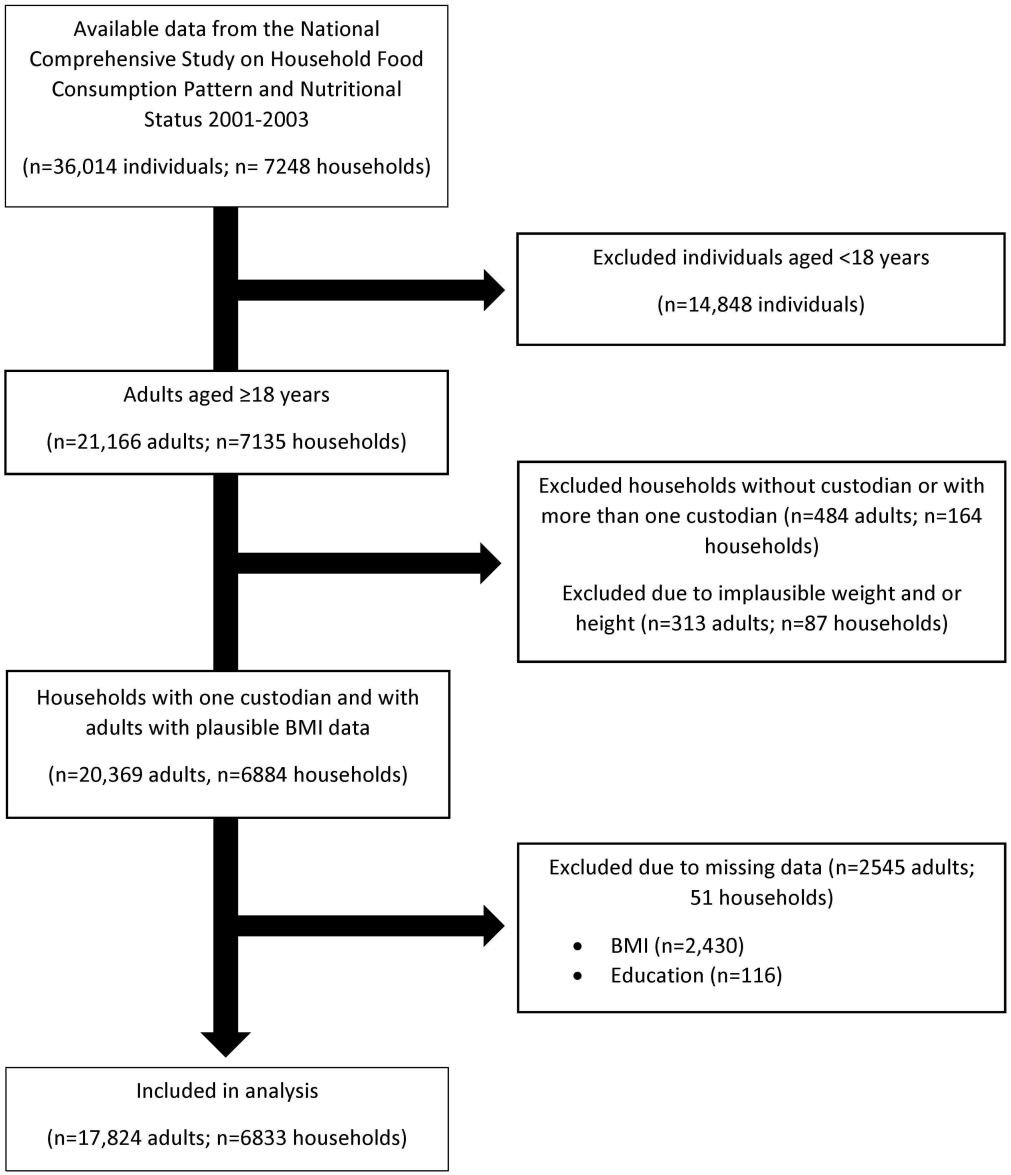
Participants flow diagram.

Of the 36,014 individuals (*n* = 7,248 households) with available data from the National Comprehensive Study on Household Food Consumption Pattern and Nutritional Status 2001–2003, 797 adults were excluded for being ineligible (implausible weight and height and 0 or > 1 custodian) and 2,545 adults were excluded for missing data (Figure 1). A total of 17,824 adults were included in the present study. The mean age of adults was 37.3 (15.1) years, the mean energy intake of adults was 2,660 (692.27) kcal, and mean weight and BMI were 66.2 (13.7) kg and 25.2 (5.0) kg/m^2^, respectively. Forty seven percent were overweight or obese. As shown in Table 1, the majority of adults were young (18–40 years; 62%), female (55%), had low education (50%) and lived in urban areas (65%), with a mean energy intake of 2,660 (696) kcal/day. At the household level, the mean age of household heads was 45.5 (13.3) years and the mean energy intake of household heads was 2,635 (694.45) kcal. The majority of household heads were males (95%), middle aged (44%), lived in urban areas (65%), and had low levels of education (57%).

**Table 1:** Characteristics of Iranian adults overall and by household heads.

Characteristics	Overall, *n* = 17,824 adults *N* (%) or Mean (SD)	Household Heads, *n* = 6,833 households *N* (%) or Mean (SD)
Age^1^, *N* (%)		
Young age	11,041 (61.94)	2,736 (40.04)
Middle-aged	4,463 (25.04)	2,979 (43.60)
Older	2,320 (13.02)	1,118 (16.36)
Sex, *N* (%)		
Males	8,067 (45.30)	6,501 (95.14)
Females	9,757 (54.74)	332 (4.86)
Level of education^2^, *N* (%)		
Low	8,891 (50.00)	3,869 (56.62)
Moderate	7,220 (40.51)	2,312 (33.84)
High	1,713 (9.61)	652 (9.54)
Area of residence, *N* (%)		
Rural	6,289 (35.30)	2,386 (34.92)
Urban	11,535 (64.72)	4,447 (65.08)
Anthropometrics, mean (SD), *N* (%)^3^		
Overweight/obesity, *N* (%)	8,381 (47.02)	3,999 (58.52)
Weight (kg)	66.2 (13.69)	71.1 (13.41)
BMI (kg/m^2^)	25.2 (5.02)	25.2 (4.34)
Energy intake (kcal), mean (SD)	2,660 (692.27)	2,635 (694.45)

1 Young aged (18- ≤ 40 years old), middle aged (>40- ≤ 60 years old), older (>60 years old).

2 Low (no formal schooling, less than primary school, the primary school completed), medium (secondary school completed, high school completed), and high (college/university completed).

3 Data on anthropometrics were available for 5,666 household heads only.

**Figure 2:**
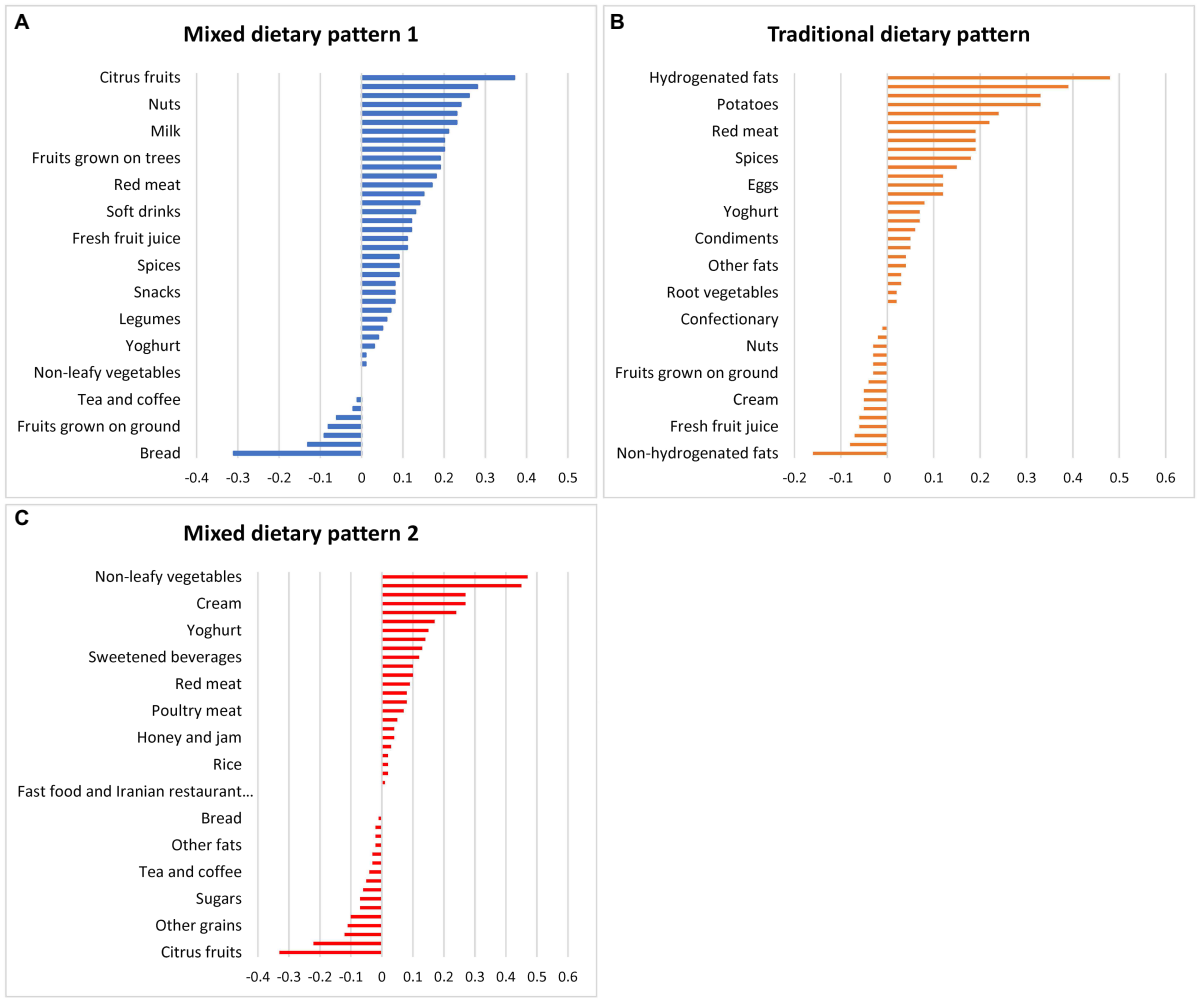
Factor loadings for the **(A)** mixed dietary pattern 1, **(B)** traditional dietary pattern, and **(C)** mixed dietary pattern 2 in Iranian household identified by principal component analysis.

Three dietary patterns were extracted using PCA. Factor loadings for the dietary patterns are presented in Figure 2. The first dietary pattern, referred to as mixed dietary pattern 1, was characterized by higher intake of citrus fruits, leafy vegetables, non-hydrogenated fats, nuts, root vegetables, rice, milk, cakes and poultry meat and low intake of breads. The second dietary pattern, referred to as traditional dietary pattern, was characterized by higher intake of hydrogenated fats, onions, potatoes, tomato pastes, legumes and breads. The third dietary pattern, referred to as mixed dietary pattern 2, was characterized by higher intake of non-leafy vegetables, fruits grown on the ground, soft drinks, cream and fruits grown on trees and low intake of citrus fruits and legumes. Nutrient intake (mean and standard error) and diet quality indices according to tertiles of each dietary pattern are presented in Table 2. Energy intake was significantly higher with increasing tertiles of the traditional dietary pattern. In contrast, energy intake was lower in the second tertile of the mixed dietary patterns, and higher in the third tertile. Intake of sodium, calcium, fiber, and vitamin C were higher across tertiles of all dietary patterns, while iron intake was higher across tertiles of the mixed dietary pattern 1 and traditional dietary pattern. The percentage of energy from fat was higher across tertiles of all three dietary patterns, while the percentage of energy from carbohydrates was lower. The percentage of energy from protein was higher across tertiles of mixed dietary pattern 1 but was lower across tertiles of the traditional dietary pattern. The mean HEI score was higher across tertiles of the mixed dietary patterns while the mean DQI-I score was higher across all three dietary patterns.

**Table 2:** Household nutrient intakes and diet quality indices according to tertiles of dietary patterns (*n* = 6,833 households)^1^.

	Mixed dietary pattern 1			Traditional dietary pattern			Mixed dietary pattern 2		
	T1	T2	T3	T1	T2	T3	T1	T2	T3
Nutrients									
Protein (%E)	10.74 (0.0)	10.96 (0.0)	11.48 (0.0)	11.4 (0.0)	11.0 (0.0)	10.8 (0.0)	11.08 (0.0)	11.06 (0.0)	11.07 (0.0) *
Fat (%E)	21.10 (0.2)	24.85 (0.1)	27.50 (0.2)	22.9 (0.2)	24.1 (0.1)	26.8 (0.2)	22.34 (0.2)	24.41 (0.2)	27.00 (0.2)
Carbohydrate (%E)	68.69 (0.1)	64.66 (0.1)	61.54 (0.2)	66.2 (0.2)	65.4 (0.1)	62.10 (0.2)	67.07 (0.2)	65.00 (0.2)	62.56 (0.2)
Sodium (mg/day)	1,730 (34.8)	2,196 (44.2)	2,812 (70.1)	1,649 (24.7)	2,112 (33.2)	3,042 (81.4)	2,084 (41.0)	2,147 (53.7)	2,551 (63.6)
Calcium (mg/day)	521.80 (4.8)	551.50 (4.4)	698.1 (5.4)	518.25 (4.5)	579.72 (4.7)	683.70 (5.6)	573.23 (5.2)	564.30 (4.8)	641.04 (5.4)
Iron (mg/day)	15.02 (0.1)	14.23 (0.1)	15.61 (0.1)	12.18 (0.1)	14.65 (0.1)	18.13 (0.1)	15.30 (0.1)	14.43 (0.1)	15.17 (0.1) *
Fiber (g/day)	11.2 (0.1)	11.3 (0.1)	13.1 (0.1)	9.5 (0.1)	11.6 (0.1)	14.7 (0.1)	11.90 (0.1)	11.30 (0.1)	12.55 (0.1)
Vitamin C (mg/day)	35.8 (0.7)	54.6 (0.7)	93.6 (1.2)	51.12 (0.9)	60.00 (0.9)	75.93 (1.1)	61.82 (1.2)	49.78 (0.8)	74.70 (1.0)
Energy intake (kcal)	2,611(15.54)	2,530 (13.27)	2,758 (14.41)	2,136 (10.18)	2,580 (10.06)	3,202 (13.30)	2,611 (14.96)	2,563( 14.25)	2,728 (14.21)
Diet quality indices									
HEI	29.9 (0.11)	33.9 (0.12)	37.5 (0.13)	34.5 (0.14)	33.8 (0.13)	33.5 (0.13)	32.7 (0.14)	33.1 (0.12)	35.9 (0.13)
DQI-I	37.2 (0.14)	36.5 (0.16)	39.3 (0.17)	36.5 (0.15)	37.6 (0.16)	39.0 (0.17)	36.9 (0.15)	36.7 (0.15)	39.5 (0.17)

Abbreviations: HEI, healthy eating index; DQI-I, diet quality index international

1 Wald test in unadjusted linear regression analysis for the association between nutrient intakes (continuous dependent) and tertiles of dietary patterns (categorical independent). Values represent mean and standard errors.

* The trends of nutrient intakes and diet quality indices across dietary pattern tertiles are significant *p* < 0.001 apart from protein and iron intake across third dietary pattern.

The mixed dietary pattern 1 and traditional dietary pattern were associated with household heads with higher education levels and living in urban areas, while the traditional dietary pattern was associated with household heads with lower education levels and living in rural areas (Table 3). The Iranian Food Composition Dataset was used to evaluate the percentage of energy contribution from each food group used in the PCA. The food groups that contributed most to energy intakes were breads (36.7%), rice (14.7%), hydrogenated fats (12.5%), and sugars (7.4%) (Supplementary Table 3).

**Table 3:** Associations between sociodemographic characteristics of household heads and principal component dietary patterns (n = 6,833 households).

Characteristics	Mixed dietary pattern 1^4^			Traditional dietary pattern^4^			Mixed dietary pattern 2^4^		
	β - Coeff	95% CI	*p*-Value^3^	β - Coeff	95% CI	*p*-Value^3^	β - Coeff	95% CI	*p*-Value^3^
Age of household head^1^									
Young age (reference)	-	-	-	-	-	-	-	-	-
Middle-aged	−0.08	−0.20, 0.04	0.18	−0.01	−0.13, 0.11	0.89	0.04	−0.08, 0.15	0.54
Older	−0.21	−0.41, 0.03	0.09	0.13	−0.12, 0.37	0.30	−0.05	−0.27, 0.17	0.64
Sex of household head									
Males (reference)	-	-	-	-	-	-			
Females	−0.03	−0.18, 0.13	0.73	0.04	−0.12, 0.19	0.63	0.10	−0.04, 0.25	0.15
Level of education of household head^2^									
Low (reference)	-	-	-	-	-	-	-	-	-
Moderate	0.90	0.82, 0.98	<0.001	−0.04	−0.12, 0.04	0.31	0.42	0.34, 0.49	<0.001
High	1.46	1.34, 1.58	<0.001	−0.19	−0.31, −0.07	0.002	0.68	0.57, 0.79	<0.001
Area of residence									
Rural (reference)	-	-	-	-	-	-	-	-	-
Urban	0.57	0.50, 0.64	<0.001	−0.24	−0.31, −0.17	<0.001	0.33	0.27, 0.40	<0.001

1 Young aged (18- ≤ 40 years old), middle aged (>40- ≤ 60 years old), older (>60 years old).

2 Low (no formal schooling, less than primary school, the primary school completed), medium (secondary school completed, high school completed), and high (college/university completed).

3 Wald test in linear regression analysis for the association between sociodemographic characteristics (categorical independent) and dietary patterns (continuous dependent) were adjusted for age, sex, education of household head, and area of residence.

4 The first dietary pattern was characterized by higher intake of citrus fruits, non-hydrogenated fats, leafy and root vegetables, rice and low intake of breads. The second dietary pattern was characterized by higher intake of hydrogenated fats, onions, potatoes, legumes and breads. The third dietary pattern was characterized by higher intake of fruits grown on trees, cream, soft drink, and lower intake of citrus fruits and legumes.

The adjusted intakes of food groups used in the PCA are presented stratified by weight status (non-overweight/obesity, overweight/obesity) in Supplementary Table 4. While intakes of breads and sugars intake were significantly higher in non-overweight/obese groups, the intake of all other food groups (rice, other grains, leafy vegetables, non-leafy vegetables, tomato paste, onions, root vegetables, citrus fruits, fruits grown on trees, fresh fruit juice, dried fruits, red meat, poultry meat, processed meats, fish and sea foods, eggs, cheese, cream, non-hydrogenated fats, cakes and desserts, sweet biscuits, soft drinks, sweetened beverages, condiments, and spices) was significantly higher in adults with overweight/obesity than adults with non-overweight/obesity.

The association between household dietary patterns and individual-level BMI and obesity are presented in Table 4. All three dietary patterns were positively associated with BMI. A one-unit increase in mixed dietary pattern 1, the traditional dietary pattern and mixed dietary patterns 2 was associated with a 0.49, 0.17, and 0.17 -unit increase in BMI, respectively. Furthermore, households with higher scores for all three dietary patterns were more likely to be overweight/obese.

**Table 4:** Associations between household principal component dietary patterns and adult BMI (continuous and binary) overall and by area of residence (*n* = 17,824 adults).

Dietary patterns	BMI			Overweight/Obesity^1^		
	β - Coeff	95% CI	*p*-Value^2^	OR	95% CI	*p*-Value^3^
Mixed dietary pattern 1						
Overall	0.49	0.43, 0.55	<0.001	1.28	1.24, 1.32	<0.001
Rural (*n* = 6,289)	0.71	0.61, 0.82	<0.001	1.40	1.35, 1.52	<0.001
Urban (*n* = 11,535)	0.38	0.31, 0.45	<0.001	1.20	1.17, 1.26	<0.001
Traditional dietary pattern						
Overall	0.17	0.09, 0.25	<0.001	1.08	1.04, 1.13	<0.001
Rural (*n* = 6,289)	0.33	0.18, 0.47	<0.001	1.20	1.11, 1.29	<0.001
Urban (*n* = 11,535)	0.08	−0.02, 0.18	0.105	1.02	0.97, 1.08	0.36
Mixed dietary pattern 2						
Overall	0.17	0.11, 0.24	<0.001	1.07	1.04, 1.11	<0.001
Rural (*n* = 6,289)	0.16	0.04, 0.28	0.010	1.08	1.01, 1.15	0.025
Urban (*n* = 11,535)	0.17	0.10, 0.25	<0.001	1.07	1.03, 1.11	0.001

1 BMI≥ 25 kg/m^2^.

2 Wald test in multi-level linear regression analysis for the association between dietary patterns (continuous independent) and BMI (continuous dependent) adjusted for age (continuous), sex, education, area of residence and energy intake in adults, with a mean energy intake of 2,660 kcal (SD 696).

3 Wald test in multi-level logistic regression analysis for the association between dietary patterns (continuous independent) and obesity (categorical dependent), adjusted for age, sex, education, area of residence and energy in adults; non-obese considered as reference group.

The direction of associations was comparable for all patterns after adjusting models for the ratio of energy intake: energy requirement, instead of energy intake. However, the strength of the association for the traditional dietary pattern became larger (mixed dietary pattern 1: β 0.57, 95% CI: 0.52, 0.63; traditional dietary pattern: β 0.69, 95% CI: 0.62, 0.77; mixed dietary pattern 2: β 0.24, 95% CI: 0.17, 0.30). Furthermore, results showed that 29, 46, and 25% of adults came from households with no children, less than three children, and more than three children, respectively. The direction and strength of associations with BMI were comparable for all patterns after adjusting for household size: mixed dietary pattern 1: β 0.50, 95% CI: 0.44, 0.56; traditional dietary pattern: β 0.17, 95% CI: 0.09, 0.25; mixed dietary pattern 2: β 0.17, 95% CI: 0.11, 0.24.

Tests for interaction by area of residence and sex showed a significant interaction for area of residence on the association between mixed dietary pattern 1 and BMI (P-interaction ≤0.001) and overweight/obesity (P-interaction = 0.001), while the interactions for area of residence on the association between the traditional dietary pattern and mixed dietary pattern 2 and BMI and overweight/obesity were not significant (interaction *p*-values all >0.05). Sex did not modify the association between dietary patterns and BMI and overweight/obesity (interaction p-values all >0.05). As shown in Table 4, findings by area of residence for BMI and overweight/obesity showed strong evidence for larger effect sizes in rural areas for mixed dietary pattern 1.

## Discussion

The aim of this study was to examine the association between PCA-derived dietary patterns and sociodemographic characteristics and overweight and obesity in Iranian adults. Three dietary patterns were identified that demonstrated unique characteristics. The first and third dietary patterns were mixed, including both healthier and unhealthier foods, whereas the second dietary pattern was closer to a traditional Iranian dietary pattern. In turn, the mixed dietary patterns were associated with higher education and living in urban areas while the traditional pattern was associated with lower levels of education and living in rural areas. While all three dietary patterns were positively associated with obesity, mixed dietary pattern 1 showed the strongest association. Furthermore, the association was stronger in rural areas for mixed dietary pattern 1. This study provides insights into culturally-specific dietary patterns in a nationally representative sample of Iranian adults and will help inform the design of population-level dietary interventions and policies to address obesity.

The traditional dietary pattern identified in this study is similar to an Iranian dietary pattern identified by Esmaillzadeh et al. (17). The diet of Middle-Eastern countries, including Iran, is characterized by high consumption of refined grains and hydrogenated fats (17). These foods were observed in the traditional dietary pattern, which was comprised of higher amounts of hydrogenated fats, onions, potatoes, tomato pastes, legumes and breads. In addition, higher intake of this pattern was associated with the highest intake of iron and sodium of all three patterns. This may reflect the highest factor loading for intake of red meat in this pattern, as well as national data on sodium intake, where it is estimated that 41% of the Iranian population consume salt at least two times more than the WHO recommended level (42). However, Iran, like other Middle-Eastern countries, has experienced a nutrition transition over the last three decades and diets have shifted towards unhealthier dietary patterns (1). Some unhealthier foods found in Western and unhealthy dietary patterns in other studies were also observed in the present study (17, 19). For example, mixed dietary pattern 1 included high intake of cakes, and the mixed dietary pattern 2 included high intake of soft drinks and creams and low intake of legumes and citrus fruits. This is comparable to a recent study that examined trends in dietary patterns of 6,508 Iranian adults aged 18 years and over from Tehran. A mixed dietary pattern, characterized by higher simple sugars intake was identified, for which adherence increased from 29.7 to 34.1% between the years 2006 to 2017 (43).

No studies have examined associations between overall dietary patterns and area of residence in Iran. In terms of associations with specific foods, previous research is inconsistent. While a study reported higher consumption of fruits and vegetables in provinces with higher rates of urbanization (44), another study did not find any difference in fruits and vegetable intake between rural and urban areas (45). The present study identified that the mixed dietary patterns were associated with living in urban areas, while the traditional dietary pattern was associated with living in rural areas. While the nutrition transition has been occurring across Iran, these findings, and that of others, suggest that rural regions may be more inclined to uphold traditional dietary habits (46). This may be due to limited availability of Western foods in rural areas, or differences in cultural and socio-economic determinants of diet, where adults who reside in rural areas are older and more inclined to prepare traditional meals (47). However, given no studies have examined dietary patterns in relation to area of residence, and this study identified stronger associations between mixed dietary pattern 1 and obesity in rural areas, prospective research is needed to confirm these findings and to determine which dietary factors should be targeted as part of obesity prevention interventions in these population groups.

The existing evidence for associations between dietary patterns and age, sex and education are mixed (15, 16, 18, 20–22). In line with our study, previous studies have found no evidence of associations between age and sex and dietary patterns (16, 18), however, other studies in Iranian populations suggest positive associations between age, being female and consuming a healthy dietary pattern (15, 20–22). A likely explanation for the lack of associations observed in this study is that age and sex of household head were assessed, where over 95% of household heads were male and were mostly middle-aged, compared to 5% female and mostly of younger age in the overall sample. As females are more likely to be the food providers in Middle Eastern countries, and males may be more likely to consume foods outside of the home, further research is needed to quantify the individual-level sociodemographic correlates of dietary patterns in Iran (15, 48, 49).

The current findings showed that all three dietary patterns were positively associated with BMI and overweight and obesity, despite being characterized by both healthy and unhealthy food groups and higher overall diet quality. All three dietary patterns comprise both healthy and less healthy food groups and share some similarities, however each had some unique characteristics. For example, the mixed dietary pattern 1 comprises components including cakes and desserts, the traditional dietary pattern includes hydrogenated fats and the mixed dietary pattern 2 consists of cream and soft drink. Evidence for associations between dietary patterns and BMI and obesity in Iranian populations is inconsistent (12, 15–17, 22), with some studies reporting that healthier dietary patterns were associated with lower BMI (12, 16, 17), and others reporting that higher consumption of an unhealthier dietary pattern was associated with lower BMI and prevalence of obesity (15, 22). The large variation in sample size and representativeness in previous research may explain these differences, ranging from 10,693 adults from Yazd (12), 418 adults from Shiraz (22), 267 adults from Tehran (16), 486 women from Tehran (17) and 4,834 adults from Isfahan (15). In addition to the distinct sample differences, another possible explanation for these discrepancies could be the measurement of dietary intake and the availability of physical activity data. While this study collected dietary data at the household level, other studies measured dietary intakes at the individual level (12, 15–17, 22). Moreover, the present study did not adjust for physical activity, yet other studies did (12, 16, 17). Thus, the positive associations with BMI identified in this study should be interpreted with this in mind. Furthermore, this study has revealed that 47% of adults had overweight/obesity in 2002–2003, which was comparable to evidence from 2005 that showed the prevalence of overweight/obesity was 43 and 52% in Iranian men and women, respectively (50). However, the most recent national data on overweight and obesity in Iran (2016) indicates that the prevalence of overweight/obesity has risen to 59%, thus future research is needed to determine if associations between dietary patterns and BMI persist in more recent national data (3). Nevertheless, the mixed dietary patterns identified in the current study was similar to the unhealthy dietary patterns identified by previous studies. These dietary patterns were high in soft drinks, red meat, confectionary, and vegetable oils and were positively associated with obesity (16, 17, 19). Furthermore, high intakes of legumes, grains, and potatoes in the traditional dietary pattern of Sarkhosh-Khorasani et al. (12) are comparable to the traditional dietary pattern identified in the present study, which were both positively associated with obesity. Despite both traditional and mixed dietary patterns being positively associated with obesity, the larger effect size for dietary pattern 1 suggests that a more Western dietary pattern, particularly one high in soft drinks and low in vegetables, is most detrimental to risk of obesity. The possible explanation may be that, while all models were adjusted for total energy intake, this association was driven by high consumption of energy-dense foods and beverages. This dietary pattern was the only one characterized by intake of soft drinks, and contributed to the highest percentage of energy from total fat. Given the percentage from saturated fats and added sugars was not available, it was not possible to determine whether these were highest in this dietary pattern, thus this warrants further investigation.

This study has several strengths. This study included a nationally representative sample, which allowed us to examine dietary patterns across key demographic variables. Three 24-h dietary recalls were used to collect data on foods and beverages consumed in the previous 24 h. These were collected in every province throughout the year (except for Ramadan and New Year public holiday period) to capture seasonal variation in dietary intake. Variation was also captured throughout the week by including week days and weekends. The 24-h dietary recall interview required at least 20 min to be completed and could be administered through a phone call or a face-to-face interview by a trained interviewer. Since trained interviewers administered the questionnaires, literate respondents were not required, and as the recall period was over the last 24-h, respondents are likely to mostly recall what they have consumed. However, recall biases may still be present (51–53). With limited information available regarding the characteristics of dietary patterns nationally and in rural areas, this study addresses this gap. In addition, this study uses a statistical dietary pattern method that creates patterns based on foods that are commonly consumed together, thus enabling investigation of culturally-specific combinations of foods as part of an overall dietary pattern.

This study acknowledges several limitations that should be considered in the interpretation of the present findings. Firstly, the household dietary data is likely to be less precise than individual dietary data because it assumes that each household member has consumed the foods relative to his/her energy and nutrient requirements (31). Although adjustment for household size did not change the direction or strength of the associations, the impact of excluding children when assessing household-level dietary data warrants further investigation. Secondly, the cross-sectional study design is susceptible to reverse causality when examining the association between dietary patterns and overweight and obesity, thus future prospective research is needed to confirm our findings. Thirdly, one of the limitations of studies conducted in the Middle East is the lack of data on alcohol intake (54). The present study did not collect data on alcohol intake, physical activity and smoking habits, which may be important confounders and should be considered in the interpretation of associations between dietary patterns and overweight and obesity. Fourthly, while the number of fast-foods outlets is likely to have increased in Iran over recent decades, data on foods consumed out of the home were not collected in this survey. This could be important for examining dietary patterns of Iranians in the advent of more Westernized diets. Fifthly, as the Iranian population, like other Middle Eastern countries, is susceptible to central obesity and the complications of this type of obesity (55), further research is needed to measure both waist circumference and BMI. Sixthly, there was evidence of underreporting in dietary intake as the effect size for the association between the traditional dietary pattern and BMI increased after adjusting for the ratio of energy intake to requirement. The lower levels of education and adults living in rural areas may be proxies for underreporting of energy intake as our results showed positive associations between these sociodemographic characteristics and the traditional dietary pattern. Seventhly, 2% of households had only two recalls, which may impact on the variety of foods consumed over two rather than 3 days. Lastly, although this study used data collected in 2001–2003, this period of time marks the beginning of the nutrition transition in Iran (2). Thus, this study will be important for future research aiming to understand how diets have changed during the nutrition transition. Moreover, as most research in national Iranian data has focused on single foods and nutrients (27), this study addressed an important gap by providing the most recent evidence on the dietary patterns of a national sample of Iranians.

## Conclusion

The present study identified three distinct PCA-derived dietary patterns in a large and nationally representative sample of Iranian adults. While all three patterns were associated with higher BMI and odds of overweight and obesity, the socio-demographic characteristics of Iranian adults who consumed them were different. As Iran is undergoing a nutrition transition, future research is needed to examine dietary patterns at the individual level, in both rural and urban areas of Iran. As all dietary patterns identified were positively associated with obesity, further research in Iranian adults is required to identify the foods that characterize a healthful dietary pattern and are protective with respect to obesity. Moreover, analysis of are prospective cohorts that include relevant confounding factors is needed to be able to understand and compare the current results.

## Data availability statement

The original contributions presented in the study are included in the article/Supplementary material, further inquiries can be directed to the corresponding author.

## Ethics statement

The studies involving human participants were reviewed and approved by the Ethics Committee of Shahid Beheshti University of Medical Sciences and was exempted by the Deakin University Human Research Ethics Committee (reference number 2019-288). Written informed consent to participate in this study was provided by the participants’ legal guardian/next of kin.

## Author contributions

MA and AH designed and carried out the National Comprehensive Study on Household Food Consumption Pattern and Nutritional Status 2001–2003. SE, RL, SM, and KL designed the analysis. SE conducted the statistical analysis and drafted the manuscript. All authors contributed to the article and approved the submitted version.

## Funding

KL is supported by a National Health and Medical Research Council Emerging Leadership Fellowship (APP1173803). RL was supported by a National Heart Foundation Postdoctoral Research Fellowship (ID102109) and is currently supported a National Health and Medical Research Council Emerging Leadership Fellowship (APP1175250).

## Conflict of interest

The authors declare that the research was conducted in the absence of any commercial or financial relationships that could be construed as a potential conflict of interest.

## Publisher’s note

All claims expressed in this article are solely those of the authors and do not necessarily represent those of their affiliated organizations, or those of the publisher, the editors and the reviewers. Any product that may be evaluated in this article, or claim that may be made by its manufacturer, is not guaranteed or endorsed by the publisher.

## Abbreviations

BMI: Body mass index
DAG: Directed acyclic graph
DQI-I: Diet quality index-international
HEI: Healthy eating index
PCA: Principal Component analysis
WHO: World Health Organization
