# Supplementary material for: Dietary patterns derived using principal component analysis and associations with sociodemographic characteristics and overweight and obesity: A cross-sectional analysis of Iranian adults
Source: Front Nutr. 2023 Apr 17;10:1091555. doi: 10.3389/fnut.2023.1091555 (PMC10149977; doi:10.3389/fnut.2023.1091555)
Supplement: Supplementary file 1 [file Data_Sheet_1.zip › Supplementary Material/Supplementary Figure 1.DOCX]

**Supplementary Figure 1**. Directed acyclic graph of associations between dietary patterns and obesity
