# Supplementary material for: Dietary patterns derived using principal component analysis and associations with sociodemographic characteristics and overweight and obesity: A cross-sectional analysis of Iranian adults
Source: Front Nutr. 2023 Apr 17;10:1091555. doi: 10.3389/fnut.2023.1091555 (PMC10149977; doi:10.3389/fnut.2023.1091555)
Supplement: Supplementary file 1 [file Data_Sheet_1.zip › Supplementary Material/Supplementary Table 1.DOCX]

**Supplementary Table 1.** Strengthening the Reporting of Observational Studies in Epidemiology—Nutritional Epidemiology (STROBE-nut)

| **Item** | **Item nr** | **Reported on page #** |
| --- | --- | --- |
| **Title and abstract** | 1 | 1, 2 |
| **Introduction** |  |  |
| Background, rationale | 2 | 3 |
| Objectives | 3 | 4 |
| **Methods** |  |  |
| Study design | 4 | 4 |
| Settings | 5 | 4 |
| Participants | 6 | 4 |
| Variables | 7 | 5-7 |
| Data sources - measurements | 8 | 4-7 |
| Bias | NA | NA |
| Study Size | 9 | 3 |
| Quantitative variables | 10 | 5-7 |
| Statistical Methods | 11 | 7-8 |
| **Results** |  |  |
| Participants | 12 | 9 and figure 1 |
| Descriptive data | 13 | 9 and Table 1 |
| Outcome data | 14 | 9-11 and Tables |
| Main results | 15 | 9-11 and Tables |
| Other analyses | NA | NA |
| **Discussion** |  |  |
| Key results | 16 | 11, 12 |
| Limitation | 17 | 14 |
| Interpretation | 18 | 11-16 |
| Generalizability | 19 | 11-16 |
| **Other information** |  |  |
| Funding | 20 | 17 |
| Ethics | 21 | 5 |
| Supplementary material | 22 | Separate document |
