# Supplementary material for: Dietary patterns derived using principal component analysis and associations with sociodemographic characteristics and overweight and obesity: A cross-sectional analysis of Iranian adults
Source: Front Nutr. 2023 Apr 17;10:1091555. doi: 10.3389/fnut.2023.1091555 (PMC10149977; doi:10.3389/fnut.2023.1091555)
Supplement: Supplementary file 1 [file Data_Sheet_1.zip › Supplementary Material/Supplementary Table 2.DOCX]

**Supplementary Table 2**. Food groups (n=43) and their food items used in principal component analysis to derive dietary patterns

| **Food categories** | **Food groups (used in PCA)** | **Food items included** |
| --- | --- | --- |
| Cereals (4 groups) | Breads | White bread, whole grain bread, toast |
|  | Rice | White rice, rice with bran |
|  | Pasta and noodles | Pasta, noodles |
|  | Other cereals | Wheat, corn, flour and barley |
| Legumes (1 group) | Legumes | Lentils, beans, chickpeas, split chickpeas, canned legumes and mixed legumes |
| Vegetables (7 groups) | Potatoes | Red and white potato, baked potato, sweet potato, peeled potato, boiled potato |
|  | Leafy vegetables | Spinach, coriander, parsley, fenugreek, grape leaves, tarragon, lettuce, cabbage (red and white) |
|  | Dried vegetables | Spinach, leek, coriander, parsley, basil and fenugreek |
|  | Non-leafy vegetables | Cucumber, eggplant, okra, capsicum, mushroom, zucchini, tomato, green bean, green peas, olives, and canned vegetables |
|  | Tomato paste | Tomato paste |
|  | Onions | Raw, peeled onion, fried onion, boiled onion |
|  | Root vegetables | Carrot, beetroot, garlic, leek, radish |
| Fruits (5 groups) | Citrus fruits | Orange, tangerine, grapefruit, lemon |
|  | Fruits grown on ground | Melons, watermelons |
|  | Fruits grown on trees | Plum, pomegranate, fig, apple, mango, grapes, cherries, apricot, and canned fruits |
|  | Fresh fruit juice | Grape juice, apple juice, orange juice, peach juice, mango juice |
|  | Dried fruits | Dried apricot, peach, |
| Meat and alternatives (6 groups) | Red meat | lamb, beef, mixed meat and organ meat |
|  | Poultry meat | Chicken, other poultry meat and canned poultry meat |
|  | Processed meat | Sausages and salami |
|  | Fish and seafood | Fish, shrimp, other sea food and tuna |
|  | Eggs | All types of poultry eggs |
|  | Nuts | Peanut, pistachio, almond, walnut |
| Dairy products (4 groups) | Milk | Skimmed milk, high fat milk, goat milk, pasteurized milk, sterilized milk, non-pasteurized milk and curd |
|  | Yoghurt | Pasteurized yoghurt, home-made yoghurt, creamy yoghurt, Greek yoghurt and a yoghurt-based beverage |
|  | Cheese | Feta cheese, Bulgarian sheep cheese, goat cheese |
|  | Cream | Cream and ice cream |
| Fats and oils (4 groups) | Hydrogenated fats | Hydrogenated fats (solid fats) |
|  | Non-hydrogenated fats | Oils such as walnut oil, olive oil, sunflower oil |
|  | Butter | Cow, goat, sheep butter and high fat margarine |
|  | Other fats | Mayonnaise, tallow |
| Confectionary (6 groups) | Sugars | Table sugars, hard sugars (cubes) |
|  | Confectionary | Candies, chocolates, |
|  | Cakes and desserts | Sponge cakes, caramel, gelatin, desserts, donuts |
|  | Sweet biscuits | Creamy biscuits, chocolate biscuits, tea biscuits |
|  | Snacks | Chips and cheese puffs |
|  | Honey and jam | Honey, jam |
| Beverages (3 groups) | Tea and coffee | Tea and coffee |
|  | Soft drinks | Coca cola, Pepsi, Seven up, other carbonated drinks |
|  | Sweetened beverages | Fruit juice commercially prepared and fruit flavored powder drink |
| Foods out of the home (1 group) | Fast foods and Iranian restaurant dishes | Pizza, hamburgers, Iranian stew, Iranian kabab |
| Condiments (2 groups) | Condiments | Pickles, vinegar and lime juice |
|  | Spices | Turmeric, cinnamon, sumac, pepper |
