# Supplementary material for: Dietary patterns derived using principal component analysis and associations with sociodemographic characteristics and overweight and obesity: A cross-sectional analysis of Iranian adults
Source: Front Nutr. 2023 Apr 17;10:1091555. doi: 10.3389/fnut.2023.1091555 (PMC10149977; doi:10.3389/fnut.2023.1091555)
Supplement: Supplementary file 1 [file Data_Sheet_1.zip › Supplementary Material/Supplementary Table 3.PDF]

**Supplementary Table 3**: Distribution of total energy intake according to PCA food groups

| Food groups (used in PCA) | Percentage of energy (%) | Food items included |
|---|---|---|
| Breads | 36.7 | White bread, whole grain bread, toast |
| Rice | 14.7 | White rice, rice with bran |
| Pasta and noodles | 2.3 | Pasta, noodles |
| Other cereals | 0.41 | Wheat, corn, flour and barley |
| Legumes | 2.0 | Lentils, beans, chickpeas, split chickpeas, canned legumes and mixed legumes |
| Potatoes | 2.0 | Red and white potato, baked potato, sweet potato, peeled potato, boiled potato |
| Leafy vegetables | 0.61 | Spinach, coriander, parsley, fenugreek, grape leaves, tarragon, lettuce, cabbage (red and white) |
| Dried vegetables | 0.04 | Spinach, leek, coriander, parsley, basil and fenugreek |
| Non-leafy vegetables | 2.5 | Cucumber, eggplant, okra, capsicum, mushroom, zucchini, tomato, green bean, green peas, olives, and canned vegetables |
| Tomato paste | 0.54 | Tomato paste |
| Onions | 0.53 | Raw, peeled onion, fried onion, boiled onion |
| Root vegetables | 0.15 | Carrot, beetroot, garlic, leek, radish |
| Citrus fruits | 0.51 | Orange, tangerine, grapefruit, lemon |
| Fruits grown on ground | 0.55 | Melons, watermelons |
| Fruits grown on trees | 2.6 | Plum, pomegranate, fig, apple, mango, grapes, cherries, apricot, and canned fruits |
| Fresh fruit juice | 0.04 | Grape juice, apple juice, orange juice, peach juice, mango juice |
| Dried fruits* | - | Dried apricot, peach, |
| Red meat | 4.8 | lamb, beef, mixed meat and organ meat |
| Poultry meat | 1.04 | Chicken, other poultry meat and canned poultry meat |
| Processed meat | 0.45 | Sausages and salami |
| Fish and seafood | 0.33 | Fish, shrimp, other sea food and tuna |
| Eggs | 1.4 | All types of poultry eggs |
| Nuts | 0.40 | Peanut, pistachio, almond, walnut |
| Milk | 2.5 | Skimmed milk, high fat milk, goat milk, pasteurized milk, sterilized milk, non-pasteurized milk and curd |
| Yoghurt | 2.2 | Pasteurized yoghurt, home-made yoghurt, creamy yoghurt, Greek yoghurt and a yoghurt-based beverage |
| Cheese | 1.7 | Feta cheese, Bulgarian sheep cheese, goat cheese |
| Cream | 0.33 | Cream and ice cream |

| | | |
|---|---|---|
| Hydrogenated fats | 12.5 | Hydrogenated fats (solid fats) |
| Non-hydrogenated fats | 0.90 | Oils such as walnut oil, olive oil, sunflower oil |
| Butter | 0.88 | Cow, goat, sheep butter and high fat margarine |
| Other fats | 0.40 | Mayonnaise, tallow |
| Sugars | 7.4 | Table sugars, hard sugars (cubes) |
| Confectionary | 0.59 | Candies, chocolates, |
| Cakes and desserts | 0.84 | Sponge cakes, caramel, gelatin, desserts, donuts |
| Sweet biscuits | 0.37 | Creamy biscuits, chocolate biscuits, tea biscuits |
| Snacks* | - | Chips and cheese puffs |
| Honey and jam | 0.53 | Honey, jam |
| Tea and coffee* | - | Tea and coffee |
| Soft drinks | 0.30 | Coca cola, Pepsi, Seven up, other carbonated drinks |
| Sweetened beverages | 0.17 | Fruit juice commercially prepared and fruit flavored powder drink |
| Fast foods and Iranian restaurant dishes | 0.29 | Pizza, hamburgers, Iranian stew, Iranian kabab |
| Condiments | 0.11 | Pickles, vinegar and lime juice |
| Spices | 0.43 | Turmeric, cinnamon, sumac, pepper |

*Information on these food groups is not available
