# Supplementary material for: Dietary patterns derived using principal component analysis and associations with sociodemographic characteristics and overweight and obesity: A cross-sectional analysis of Iranian adults
Source: Front Nutr. 2023 Apr 17;10:1091555. doi: 10.3389/fnut.2023.1091555 (PMC10149977; doi:10.3389/fnut.2023.1091555)
Supplement: Supplementary file 1 [file Data_Sheet_1.zip › Supplementary Material/Supplementary Table 4.PDF]

Supplementary Table 4. The intake of food groups used in the PCA analyses according to weight status[1]

Food groups	Non-overweight/obesity	Overweight/obesity	P-value[2]
Bread	339.2 (2.1)	317.7 (1.9)	<0.001
Rice	106.9 (1.2)	115 (1.2)	<0.001
Pasta	16.6 (0.47)	17.0 (0.47)	0.46
Other grains	3.0 (0.17)	3.4 (0.19)	0.023
Legumes	19.0 (0.40)	18.9 (0.33)	0.98
Potatoes	57.3 (0.80)	59.0 (0.80)	0.06
Leafy vegetables	31.8 (0.66)	37.7 (0.70)	<0.001
Dried vegetables	0.45 (0.04)	0.50 (0.06)	0.43
Non-leafy vegetables	99.8 (1.5)	105.8 (1.5)	<0.001
Tomato paste	8.8 (0.14)	9.5 (0.15)	<0.001
Onions	27.0 (0.35)	28.9 (0.37)	<0.001
Root vegetables	5.2 (0.25)	6.04 (0.26)	0.004
Citrus fruits	26.4 (0.92)	36.1 (1.2)	<0.001
Fruits grown on ground	40.7 (1.5)	42.2 (1.6)	0.38
Fruits grown on trees	68.1 (1.6)	72.6 (1.5)	0.010
Fresh fruit juice	1.9 (0.28)	2.7 (0.34)	0.046
Dried fruits	1.4 (0.10)	1.60 (0.10)	0.046
Red meat	30.4 (0.56)	33.5 (0.57)	<0.001
Poultry meat	18.4 (0.47)	21.6 (0.54)	<0.001
Processed meat	4.2 (0.20)	4.7 (0.22)	0.026
Fish and seafood	6.8 (0.33)	8.3 (0.40)	<0.001
Eggs	19.6 (0.30)	21.2 (0.34)	<0.001
Nuts	2.2 (0.12)	2.4 (0.11)	0.08
Milk	37.6 (1.1)	40.0 (1.2)	0.06
Yoghurt	83.2 (1.6)	85.2 (1.5)	0.25
Cheese	14.1 (0.27)	15.3 (0.28)	<0.001
Cream	3.2 (0.19)	3.7 (0.18)	0.031
Hydrogenated fats	36.9 (0.34)	36.7 (0.33)	0.58
Non-hydrogenated fats	2.2 (0.13)	2.8 (0.15)	<0.001
Butter	3.1 (0.11)	3.1 (0.10)	0.79
Other fats	1.4 (0.11)	1.3 (0.11)	0.43
Sugars	54.3 (0.63)	47.9 (0.52)	<0.001
Confectionary	3.5 (0.18)	3.4 (0.17)	0.61
Cakes and desserts	5.2 (0.23)	6.3 (0.27)	<0.001
Sweet biscuits	1.7 (0.10)	2.1 (0.13)	0.004
Snacks	0.98 (0.09)	1.1 (0.07)	0.28
Honey and jam	5.0 (0.19)	5.3 (0.17)	0.11
Tea and coffee	4.0 (0.11)	3.8 (0.10)	0.25
Soft drinks	17.9 (0.73)	20.8 (0.89)	0.001
Sweetened beverages	2.3 (0.21)	2.8 (0.22)	0.031
Fast food and Iranian dishes	4.7 (0.40)	4.03 (0.32)	0.13
Condiments	6.1 (0.23)	7.5 (0.25)	<0.001
Spices	5.2 (0.12)	5.5 (0.10)	0.012

[1] The margin command was used to report the adjusted mean (SD) intakes of food groups used in the PCA analyses according to non-overweight/obesity (BMI<25 kg/m^2) and overweight/obesity (BMI≥25 kg/m^2)

[2] Wald test in linear regression analysis for the association between food groups (continuous independent) and BMI categories (binary dependent) adjusted for age (continuous), sex, education, area of residence and energy intake in adults
